# A neurodevelopmental disorder associated with a loss-of-function missense mutation in *RAB35*

**DOI:** 10.1016/j.jbc.2024.107124

**Published:** 2024-03-01

**Authors:** Adriana Aguila, Somaya Salah, Gopinath Kulasekaran, Moatasem Shweiki, Nava Shaul-Lotan, Hagar Mor-Shaked, Muhannad Daana, Tamar Harel, Peter S. McPherson

**Affiliations:** 1 Department of Neurology and Neurosurgery, Montreal Neurological Institute, McGill University, Montreal, Quebec, Canada; 2 Department of Genetics, Hadassah Medical Center, Jerusalem, Israel; 3 Neurosurgery Department, Hadassah Medical Center, Jerusalem, Israel; 4 Faculty of Medicine, Hebrew University of Jerusalem, Jerusalem, Israel; 5 Child Development Centers, Clalit Health Care Services, Yokne'am Illit, Israel

**Keywords:** neurodevelopmental disorder, GTPase, Rab35, recessive mutation, cytokinesis, primary cilia

## Abstract

Rab35 (Ras-associated binding protein) is a small GTPase that regulates endosomal membrane trafficking and functions in cell polarity, cytokinesis, and growth factor signaling. Altered Rab35 function contributes to progression of glioblastoma, defects in primary cilia formation, and altered cytokinesis. Here, we report a pediatric patient with global developmental delay, hydrocephalus, a Dandy–Walker malformation, axial hypotonia with peripheral hypertonia, visual problems, and conductive hearing impairment. Exome sequencing identified a homozygous missense variant in the GTPase fold of *RAB35* (c.80G>A; p.R27H) as the most likely candidate. Functional analysis of the R27H-Rab35 variant protein revealed enhanced interaction with its guanine-nucleotide exchange factor, DENND1A and decreased interaction with a known effector, MICAL1, indicating that the protein is in an inactive conformation. Cellular expression of the variant drives the activation of Arf6, a small GTPase under negative regulatory control of Rab35. Importantly, variant expression leads to delayed cytokinesis and altered length, number, and Arl13b composition of primary cilia, known factors in neurodevelopmental disease. Our findings provide evidence of altered Rab35 function as a causative factor of a neurodevelopmental disorder.

Ras-associated binding (Rab) proteins are a family of small GTPases composed of approximately 60 members in humans (1). Rabs are evolutionarily conserved and expressed in a wide variety of cell types and at multiple cellular locations (2). Rabs switch between an active GTP–bound state and an inactive GDP–bound state. While GDP-bound Rabs are primarily located in the cytosol, once GTP bound, Rabs associate with membranes *via* C-terminal prenyl groups and bind multiple effectors *via* their switch 1 and 2 regions (3). Interaction with effectors allows the Rabs to drive their plethora of membrane trafficking functions.

The exchange of GTP for GDP on Rabs is mediated by guanine-nucleotide exchange factors (GEFs). GEFs bind GDP-bound Rabs and catalyze the removal of GDP, remaining bound to the nucleotide-free form of the GTPase as an enzymatic intermediate until GTP binds the Rab, causing the GEF to dissociate. The active state of the Rab ends when the Rab GTPase hydrolyzes GTP to GDP with the catalytic aid of GTPase-activating proteins (GAPs). Rabs are evolutionarily conserved and control multiple fundamental cellular processes, such as vesicle budding and transport, tethering and fusion, organelle structure and function, lipid remodeling, and regulation of cytoskeletal dynamics (4). Rabs conduct these functions at basically all compartments in eukaryotic cells. Altered expression levels and mutations of Rab GTPases have been frequently associated with disease, including cancer, neurodegenerative diseases, and various genetic disorders (5, 6, 7, 8, 9).

Endocytosis and endosomal membrane trafficking are cellular processes regulating the localization and levels of a myriad of proteins. They are critical for the uptake and metabolism of nutrients and for the regulation of signaling cascades, by controlling the plasma membrane levels and activity of signaling receptors (10). Rab35 regulates membrane trafficking in the endosomal system (11), is broadly expressed in human tissues, and is evolutionarily conserved (12). This Rab GTPase localizes to the plasma membrane and endosomal vesicles and tubules and is implicated in endocytosis, vesicle recycling, autophagy, exocytosis, and exosome release (13, 14, 15, 16). Rab35 functions in the successful abscission of the cytokinetic bridge during cell division through its interaction with the effector oculocerebrorenal syndrome of Lowe protein (OCRL) (17). In addition, it functions in the formation and signaling of primary cilia by regulating ciliary length, function, and membrane composition (18).

Given the wide variety of cellular processes involving Rab35, altered function of the protein through mutations could result in human disease. For example, defects in cytokinesis and cilia formation have been associated with neurodevelopmental disorders (19, 20). Rab35 is required during neuronal development in the hippocampus (21), its expression is decreased in glioblastoma (22), and disruption of Rab35 enhances growth and invasiveness of human glioblastoma in mouse xenograft models (8). Here, we report a patient with a neurodevelopmental disorder carrying a variant in *RAB35* (c.80G>A; p.(Arg27His)), causing the substitution of arginine 27 (R27) for a histidine (H). The Rab35 R27H variant locks the protein in an inactive state. Cells overexpressing this variant have altered Arf6 activation and disruptions in cytokinesis and primary cilia, suggesting that the mutation is a causative factor for the abnormalities observed in the patient.

## Results

### Clinical report

**Figure 1:**
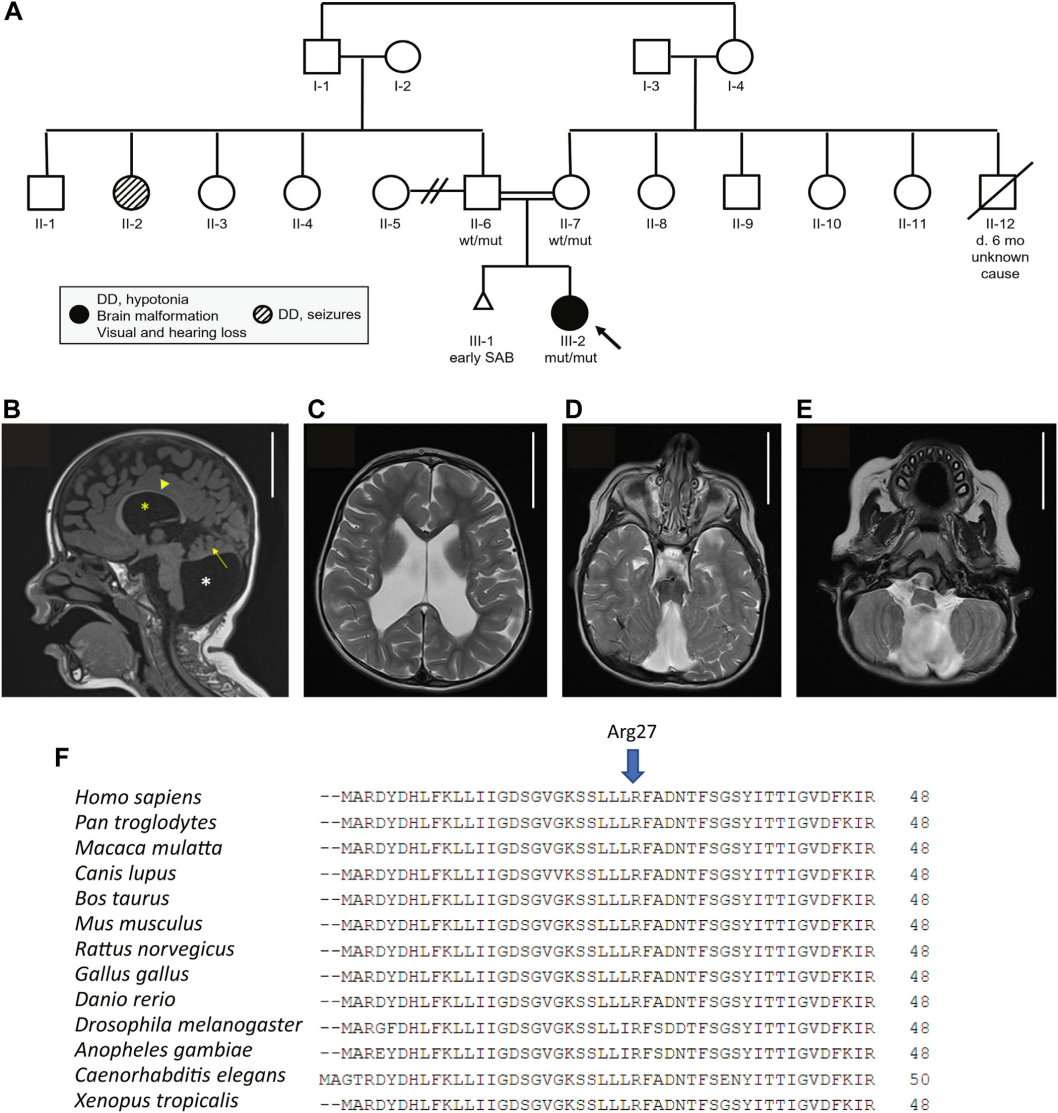
**Pedigree and clinical features of the subject.***A*, pedigree of the affected individual. The proband is III-2. Males are represented in *squares*, females in *circles*, and the *triangle* (III-1) represents a spontaneous abortion. *B*, midline sagittal T1-weighted image, indicating thinning of the corpus callosum (*yellow arrowhead*), large ventricles (*yellow asterisk*), hypoplastic cerebellar hemispheres (*yellow arrow*), aplasia of the cerebellar vermis, and mega cisterna magna (*white asterisk*). *C*, axial T2-weighted image showing enlarged ventricles. *D*, axial T2-weighted image showing agenesis of the cerebellar vermis. *E*, axial T2-weighted image demonstrating mega cisterna magna. Scale bar represents 5 cm. *F*, alignment of Rab35 from various vertebrate species revealing the conservation of the residue arginine 27 (Arg = R), the variant altered in the proband. Rab, Ras-associated binding.

The proband (Fig. 1*A*, individual III-2) was the first child of consanguineous, first cousin parents of Muslim-Arab origin, who previously had a spontaneous miscarriage. The proband was born at full term after a pregnancy complicated by maternal insulin-dependent diabetes mellitus, which was controlled before and during pregnancy. She had a birthweight of 3090 g (26th %ile) and an Apgar score of 9 at 1 min and 9 at 5 min. After birth, she had cyanosis and required oxygen treatment and had indirect hyperbilirubinemia. She was discharged from the neonatal intensive care unit at 7 days.

Developmental milestones were delayed; at 2.5 years of age, she could roll over but could not sit, crawl, or stand. She had speech delay and significant cognitive delay (developmental quotient = 32). Magnetic resonance imaging of the brain at 11 months revealed a large cyst in the posterior fossa, enlarged lateral ventricles, and a severely hypoplastic vermis, consistent with a Dandy–Walker malformation (Fig. 1, *B*–*E*). In addition, there was suspected periventricular leukomalacia indicative of white matter disease. At 35 months of age, thinning of the corpus callosum and significant loss of white matter volume were detected. The electroencephalogram was normal. Echocardiogram after birth revealed a cardiac defect that resolved spontaneously. Abdominal and renal ultrasound were normal. Moderate conductive hearing impairment and visual defects including esotropia, amblyopia, hypermetropia, astigmatism, cupping of the optic disc, and limited bilateral eye movements on abduction and upward gaze were observed. At around 2 years of age, she presented with premature thelarche. Hormonal evaluation including luteinizing hormone and follicle-stimulating hormone was normal.

Physical examination at 2.5 years of age revealed growth parameters within the normal range for age. Head circumference was 49 cm (73rd %ile), her overall length was 92.5 cm (68th %ile), and her weight was 12.8 kg (45th %ile). She had intermittent eye contact, a widely opened anterior fontanel, deep set eyes, esotropia, arched eyebrows, short philtrum, dental problems with caries, retrognathia, left-sided low set ear, abnormal palmar creases on the left hand, and mild clinodactyly of fifth fingers bilaterally. There was axial hypotonia with peripheral hypertonia. She also had hirsutism and bilateral enlargement of the breasts.

Family history was positive for insulin-dependent diabetes in the mother, with onset at approximately 16 years. A paternal aunt had global developmental delay and a seizure disorder, and a maternal uncle died at around age 6 months because of an unknown cause.

### Genetic evaluation identifies a candidate variant in *RAB35*

Genetic evaluation in the proband included a chromosomal microarray followed by trio exome sequencing, revealing a ∼130 kbp duplication (arr[GRCh37] 14q32.31(102941709_103068021)×3) of unknown clinical significance (variant of unknown significance), harboring no known triplosensitive genes. This was subsequently determined to be inherited from her unaffected father and therefore unlikely to be a major contributor to the phenotype.

Trio exome sequencing for the proband and her parents demonstrated a homozygous missense variant in *RAB35*—chr12:120546244[hg19]; NM_006861.7; c.80G>A; p.(Arg27His). Both parents are heterozygous carriers of this variant. The variant is rare (found neither in gnomAD nor in the local database of ∼14,000 exomes), affects a residue conserved in vertebrate Rab35 (GERP 4.83) (Fig. 1*F*), and is predicted to be deleterious by multiple bioinformatic tools, including MutationTaster, PolyPhen, and SIFT. Combined annotation-dependent depletion score is 24.7, further supporting pathogenicity. The encoded protein, Rab35, functions in abscission of the cytokinetic bridge (17) and in primary cilia formation and signaling (18), phenotypes associated with neurodevelopmental disorders. *RAB35* was thus considered a candidate gene leading to the disease phenotype.

Secondary findings as defined by the American College of Medical Genetics (23) included a heterozygous, likely pathogenic, variant in *RYR2* (NM_001035.3): c.11060dup; p.(Leu3687PhefsTer15) inherited from the proband's mother. Pathogenic variants in *RYR2* lead to ventricular arrhythmias (24), and therefore, the proband and her mother were referred for evaluation and follow-up with cardiology. In addition, the parents were both heterozygous for a pathogenic variant in *DARS2* (NM_018122.5): c.788G>A; p.(Arg263Gln) known to cause leukoencephalopathy with brain stem and spinal cord involvement and lactate elevation (Online Mendelian Inheritance in Man: 611105) with autosomal recessive inheritance. While the proband was not homozygous for the variant, this information has implications for prenatal testing or preimplantation genetic diagnosis in future pregnancies.

### The R27H mutation locks Rab35 in an inactive state

**Figure 2:**
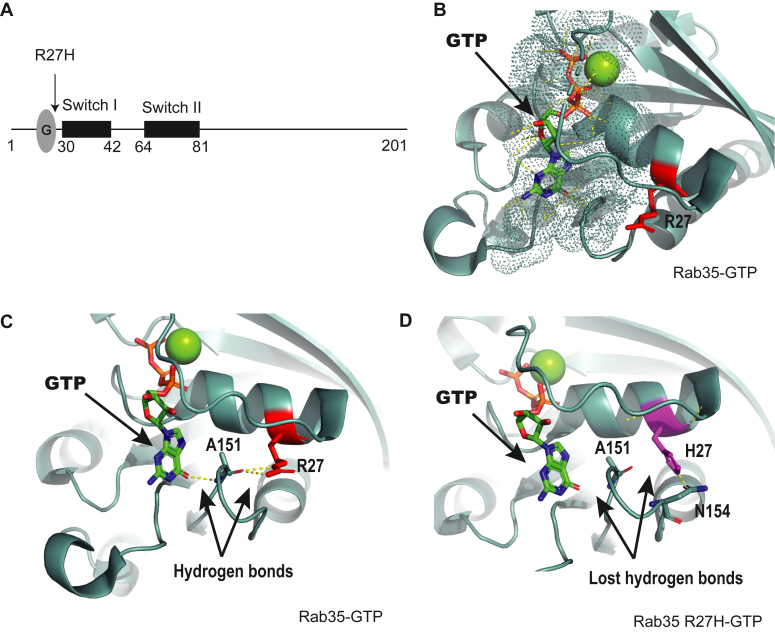
**Rab35 R27H mutation is not directly involved in nucleotide binding or interaction with its effector.***A*, schematic diagram showing the domain organizations of Rab35 and the location of the R27H mutation within the protein sequence. *B*–*D*, crystal structure of Rab35 in complex with GTP (Protein Data Bank identifier: 6IF3). *B*, the surface in contact with the nucleotide GTP is shown as *dots* and the lateral chain of arginine 27 as *sticks*. *C*, hydrogen bonds established between alanine 151 and arginine 27 residues and between alanine 151 and the guanosine ring are shown. *D*, arginine 27 residue was mutated *in silico* to a histidine residue. After curating the resulting structure, the hydrogen bond with alanine 151 was lost. Rab35 shown in *cyan*, arginine 27 shown in *red*, and histidine 27 in *magenta*. Rab, Ras-associated binding.

The R27H mutation in Rab35 is located in proximity to the G-domain (Fig. 2*A*) that is involved in nucleotide binding, but outside switch I and II regions, which are involved in interaction with effectors and GEFs (25). We examined the three-dimensional structure of Rab35 in complex with GTP (Protein Data Bank identifier: 6IF3) and recognized that the R27 is in proximity to the nucleotide-binding site but does not interact directly with the nucleotide itself (Fig. 2*B*). Interestingly, R27 interacts *via* hydrogen bonds with alanine (A) 151 of Rab35. In turn, A151 interacts with the guanosine ring of GTP (Fig. 2*C*). The R27 residue was mutated *in silico* to H27 using PyMOL and analyzed for the gain or loss of hydrogen bonds. The R27H mutation is predicted to lead to loss of the hydrogen bond with A151 (Fig. 2*D*). This in turn could destabilize the interaction between A151 and the nucleotide because of conformational changes in the protein. Thus, the R27H mutation may destabilize the nucleotide-binding site.

**Figure 3:**
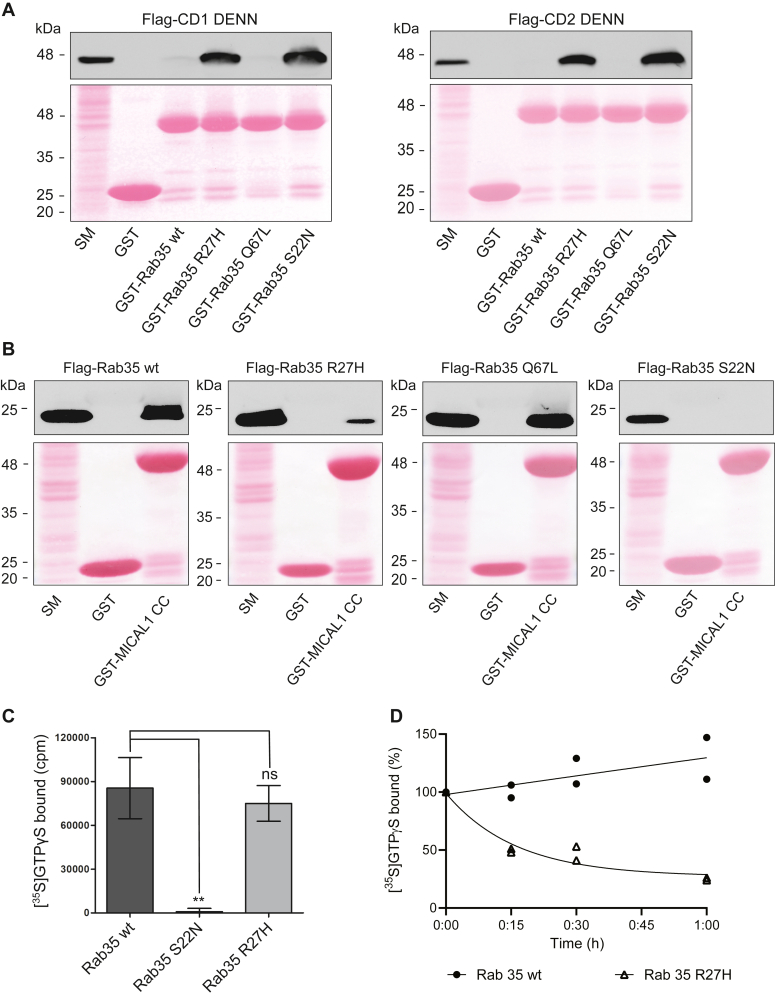
**The Rab35 R27H mutation disrupts its activation.***A*, detergent-soluble lysates were prepared from HEK-293T cells expressing FLAG-connecdenn 1 (CD1) or connecdenn 2 (CD2) DENN domains and incubated with either GST alone or GST-Rab35 wt, R27H, Q67L (active mutant), or S22N (inactive mutant). *B*, detergent-soluble lysates were prepared from HEK-293T cells expressing FLAG-Rab35 wt, R27H, Q67L, or S22N and incubated with either GST alone or GST-MICAL1 CC domain. For both assays, Glutathione-Sepharose beads were washed, and the bound proteins were prepared for immunoblot with an antibody recognizing FLAG. An aliquot of the cell lysate equal to 5% of that added to the beads was run as a starting material (SM). The Ponceau-stained transfer indicates the levels of fusion protein. *C*, *in vitro* nucleotide loading assay using purified Rab35 wt, S22N, and R27H. About 0.4 μM of purified GTPases were incubated with [^35^S]GTPγS for 10 min. Nucleotides loaded on the GTPases were stabilized by adding 5 mM MgCl_2_. The amount of [^35^S]GTPγS loaded was determined by collecting the reactions on filters, followed by scintillation counting. Data are shown as mean ± SEM. Statistical analysis employed was a one-way ANOVA followed by Dunnett’s post hoc test. ∗∗*p* < 0.01; n = 4. *D*, *in vitro* nucleotide dissociation assay using purified Rab35 wt and R27H variant. About 0.4 μM of purified GTPases were incubated with [^35^S]GTPγS for 10 min. Nucleotides loaded on the GTPases were stabilized by adding 5 mM MgCl_2_. The aliquots of reaction were diluted by adding cold wash buffer containing 20 mM MgCl_2_ and incubated for indicated time points in minutes. The amount of [^35^S]GTPγS remaining on the GTPases was determined by collecting the reactions on filters, followed by scintillation counting. The relative dissociation of [^35^S]GTPγS is plotted over time; n = 2. The curve was fitted by nonlinear regression one-phase dissociation. GST, glutathione-*S*-transferase; HEK-293T, human embryonic kidney 293T cell line; Rab, Ras-associated binding.

DENN domain proteins function as GEFs for Rabs. The connecdenn (DENND1) family members connecdenn 1–3 (DENND1A-C) exhibit GEF activity toward Rab35 (9, 11). GEFs bind their substrate GTPases in the GDP-bound inactive form (26). We thus compared Rab35 R27H, Rab35 wt, Rab35 S22N (a constitutively inactive GDP–bound mutant), and Rab35 Q67L (a constitutively active GTP–bound mutant) for binding to DENND1A/B. The DENN domains of DENND1A/B had little interaction with Rab35 wt and Q67L, whereas they interact with Rab35 S22N (Fig. 3*A*). Interestingly, the Rab35 R27H variant had a similar interaction as the S22N mutant, suggesting that Rab35 R27H is in an inactive conformation (Fig. 3*A*).

Active Rab35 interacts with effectors to mediate numerous cellular functions. We performed an effector-binding assay using a glutathione-*S*-transferase (GST)-fused coiled coil region of MICAL1 (GST-MICAL1 CC), which binds Rab35 selectively in the GTP-bound form (27). GST-MICAL1 CC binds FLAG-Rab35 wt and Q67L but neither FLAG-Rab35 R27H nor S22N, further indicating that the R27H mutation in Rab35 converts the protein to an inactive conformation (Fig. 3*B*).

Rab35 folds into an inactive conformation when GDP bound or nucleotide free. To determine if R27H alters the nucleotide-binding ability, we performed an *in vitro* nucleotide loading assay using purified Rab35 wt and the R27H variant. Surprisingly, we detected no significant difference in the GTPγS loading efficiency of Rab35 R27H compared with Rab35 wt (Fig. 3*C*). However, when Rab35 wt and the R27H variant were loaded with [^35^S]GTPγS in the presence of MgCl_2_ and the release of nucleotide was assayed by diluting the loaded GTPase, Rab35 wt displayed stable GTP interaction, whereas GTP was rapidly lost from the R27H variant (Fig. 3*D*).

### Expression of Rab35 R27H delays cytokinesis and activates Arf6

**Figure 4:**
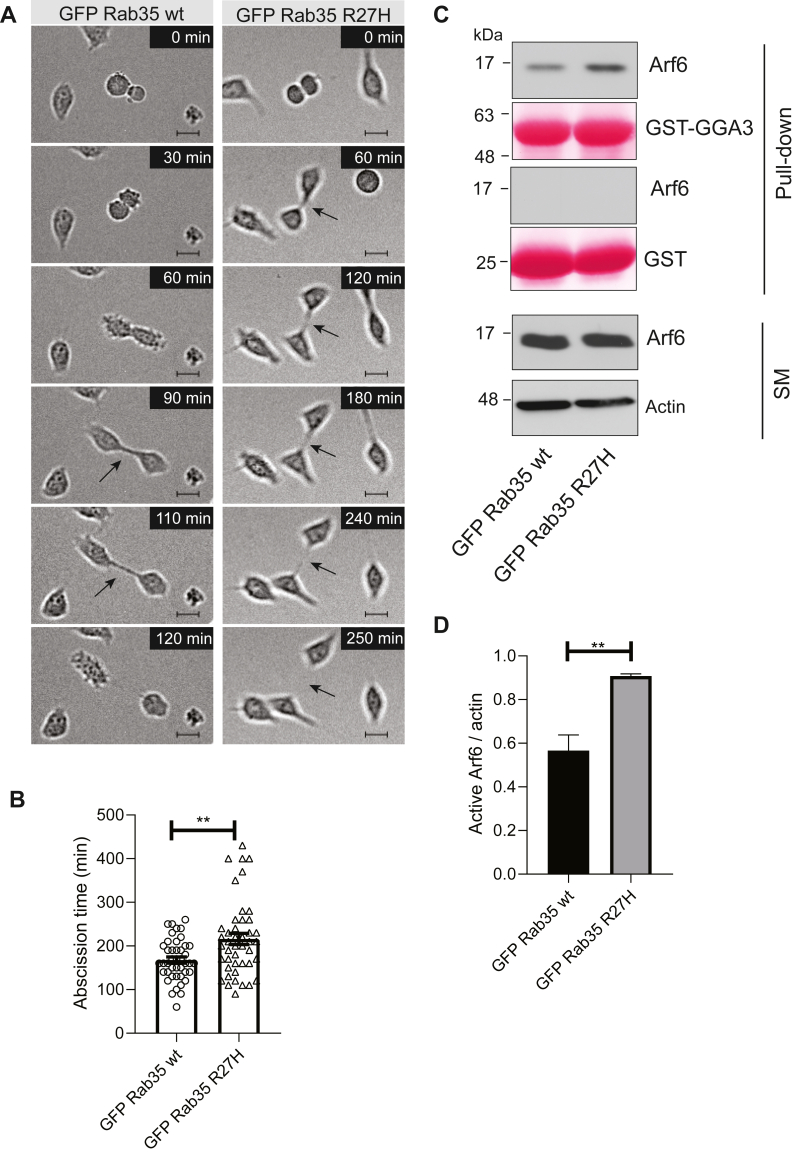
**Rab35 R27H variant overexpression delays cytokinetic bridge abscission.***A*, selected phase contrast time-lapse frames from HeLa cells transduced with lentivirus driving overexpression of either GFP-Rab35 wt or R27H, as indicated. The time at which each cell entered mitosis was marked as time “0,” and the process was considered completed with the abscission of the cytokinetic bridge. Images were taken every 10 min for 20 h. The time frame for each selected image is shown in minutes. Scale bar represents 10 μm, and *arrow* points at the cytokinetic bridge. *B*, quantification of abscission time, in minutes, from experiment described in *A*. Data are shown as mean ± SEM, n = 3, ≥43 dividing cells per experimental condition. Statistical analysis employed was Mann–Whitney *U* test; ∗∗*p* < 0.01. *C*, detergent-soluble lysates were prepared from HeLa cells transduced with lentivirus driving overexpression of either GFP-Rab35 wt or R27H and incubated with either GST alone or GST-GGA3 1 to 316 amino acids. Glutathione-Sepharose beads were washed, and the bound proteins were processed for immunoblot with an antibody recognizing Arf6. An aliquot of the cell lysate equal to 5% of that added to the beads was run as a starting material (SM). The Ponceau-stained transfer indicates the levels of fusion protein. *D*, quantification of the levels of active Arf6 relative to actin, from experiment described in *C*. Data are shown as mean ± SEM, n = 3. Statistical analysis employed unpaired *t* test; ∗∗*p* < 0.01. GST, glutathione-*S*-transferase; Rab, Ras-associated binding.

Cytokinesis is the final step of cell division and allows for the physical separation of a cell into 2 daughter cells. Membrane trafficking plays a critical role in cytokinesis (28). Rab35 functions in the stabilization and abscission of the cytokinetic bridge during cell division, in part by driving the depolymerization of F-actin at the cytokinetic bridge through recruitment of the Rab35 effectors OCRL and MICAL1 (17, 29, 30). We measured the time in which cells transduced with either GFP-Rab35 wt or GFP-R27H divided, from furrow ingression until the abscission of the cytokinetic bridge. Cells overexpressing the Rab35 R27H variant exhibited a delay in cytokinesis as compared with cells overexpressing Rab35 wt, showing an average of 216 ± 13 min to complete cytokinesis as compared with 168 ± 7.2 min, respectively (mean ± SEM) (Fig. 4, *A* and *B*).

Rab35 controls cytokinesis and the recycling of membrane receptors and adhesion proteins through mutual antagonism with Arf6. Active Rab35 drives the inactivation of Arf6 *via* ACAP2, a Rab35 effector and an Arf6 GAP (22, 31). Arf6, in turn, inhibits Rab35 *via* recruitment of TBC1D10A (32), TBC1D10B (31), and TBC1D24/Skywalker, Arf6 effectors, and Rab35 GAPs (22). To assess the levels of active Arf6 in cells overexpressing the R27H variant, we performed a pulldown assay with GST-fused GGA3, a known effector of Arf6 (33). Cells overexpressing the Rab35 R27H variant showed increased levels of active Arf6 as compared with the cells overexpressing Rab35 wt, whereas the total Arf6 levels remained the same, indicating that the R27H variant mimics results seen with Rab35 knockdown (Fig. 4, *C* and *D*).

### Rab35 R27H mutation interferes with primary cilium length and number

**Figure 5:**
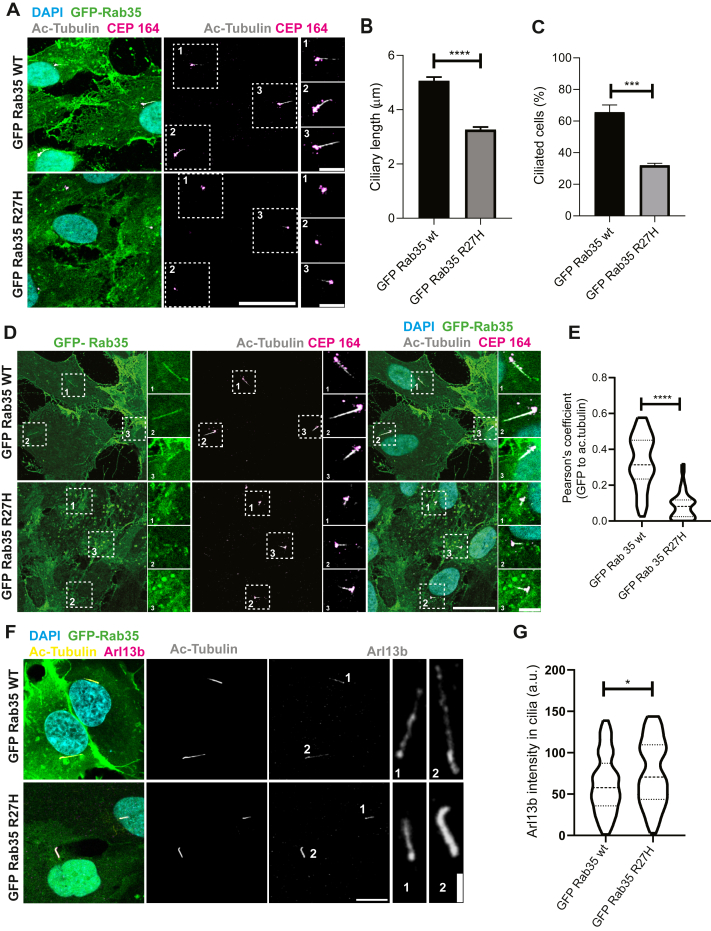
**Rab35 R27H variant affects cilia formation, length, and composition.***A*, RPE-1 cells transduced with lentivirus expressing either GFP-RAB35 wt or R27H were incubated with starvation media for 24 h. The cells were fixed in methanol and stained with antibodies recognizing CEP164 (*magenta*) and acetylated tubulin (ac-tubulin) (*gray*). DAPI (*blue*) was used to reveal nuclei. Scale bar represents 20 μm for low-magnification images. The higher magnification insets correspond to the area enclosed by *dotted squares* and are identified with numbers; scale bar represents 5 μm. *B*, quantification of ciliary length from cells treated as in *A*. *C*, quantification of percentage of ciliated cells, treated as in *A*. Data shown as mean ± SEM, n = 3, ≥187 cilia (*B*), ≥110 cells (*C*), per experimental condition. *D*, RPE-1 cells were transduced, treated, and processed as in *A*. Scale bar represents 20 μm for low-magnification images, 5 μm for higher magnification, obtained from the *dotted area*. *E*, graphical representation of the Pearson's correlation coefficient for the colocalization of GFP-Rab35 (wt or R27H) with ac-tubulin from experiment performed in *D*. Shown using a violin plot, n = 3, ≥36 cilia per experimental condition. *F*, RPE-1 cells were transduced and treated as in *A*. The cells were fixed in 4% paraformaldehyde and stained with antibodies anti-Arl13b (*magenta*) and anti-ac-tubulin (*yellow*). DAPI (*blue*) to stain nuclei. Scale bar represents 10 μm and 2 μm for *insets*, identified with numbers. *G*, quantification of the fluorescent intensity of Arl13b in the cilia, corresponding to experiment in *F*. Data are shown using a violin plot, n = 3, ≥95 cilia per experimental condition. For all quantifications, statistical analysis used was unpaired *t* test; ∗*p* < 0.05; ∗∗∗*p* < 0.001; ∗∗∗∗*p* < 0.0001. DAPI, 4′,6-diamidino-2-phenylindole; Rab, Ras-associated binding.

Rab35 functions in the formation and membrane composition of primary cilia (18). Primary cilia are microtubule-based organelles that sense and transduce environmental signals. Defects in primary cilia formation or signal transduction pathways result in abnormal neural patterning, principally in the forebrain, and leads to cerebellar hypoplasia and defective cortical neurogenesis (34). We overexpressed GFP-Rab35 R27H in RPE-1 cells, a model line for examining primary cilia formation (35). Using acetylated tubulin (ac-tubulin) as a marker for the cilia body and CEP164 for the centriole, we observed that Rab35 R27H overexpression exerts a dominant-negative effect with reduction in cilia length, which were on average, 3.3 ± 0.1 μm, whereas the cells overexpressing Rab35 wt exhibited longer cilia, with an average length of 5.1 ± 0.13 μm (mean ± SEM) (Fig. 5, *A* and *B*). The number of ciliated cells was also significantly reduced in cells overexpressing the Rab35 R27H variant (Fig. 5*C*). Rab35 is localized to the ciliary membrane when in its GTP-bound active state (18). We found that the Rab35 R27H variant is poorly localized at the cilia when compared with the wt protein (Fig. 5, *D* and *E*).

Primary cilia are involved in multiple cellular processes, and their protein composition is tightly controlled. Rab35 interacts with and regulates the levels of Arl13b at the ciliary membrane (18). Arl13b mediates the release of lipidated protein cargo and facilitates sonic hedgehog signaling (Shh) (36), a master regulator pathway of neurodevelopment (37). Because of the importance of maintaining balanced levels of Arl13b at the cilium and the involvement of Rab35 in its regulation, we studied how the R27H variant could influence Arl13b localization. We observed an increase in the ciliary levels of endogenous Arl13b in cells expressing the Rab35 R27H variant (Fig. 5, *F* and *G*), suggesting that Rab35 R27H promotes ciliary accumulation of Arl13b and affects ciliogenesis.

## Discussion

Small GTPases control membrane trafficking events crucial to the development of the central nervous system (38, 39, 40, 41), and mutations that cause loss or gain of function in small GTPases are causative of disorders featuring neurodevelopmental delay (42, 43, 44, 45). Rab35 is an abundant GTPase that controls a diverse array of cellular functions. It maintains presynaptic protein homeostasis (46), is required for axonal and neurite outgrowth (47), participates in cytokinesis (17) and primary cilia formation (4), and is an important regulator of embryonic development (48).

Here, we describe the phenotype of an individual presenting with global developmental delay, axial hypotonia and limb hypertonia, structural brain abnormalities, ophthalmic issues, and distinctive craniofacial features, in whom exome sequencing identified a homozygous missense variant in *RAB35* that results in the change of an arginine to a histidine in the 27th position. The posterior fossa abnormalities in the proband, combined with the altered primary cilia formation in cells overexpressing the missense variant (c.80G>A; p.R27H), are indicative of a ciliopathy and suggest that biallelic *RAB35* abnormalities lead to a neurodevelopmental disorder. Previous investigations have reported patients with variants of RUSC2, a Rab35 effector, which present with a neurodevelopmental disorder (49). In addition, variants in a Rab35 GAP, TBC1D24, have been associated with a spectrum of neurodevelopmental disorders in a cohort of patients (50). In this study, we demonstrate that the R27H variant limits the ability of the protein to remain in its active GTP–bound state, resulting in reduced interaction with effectors. Consequently, cellular processes coordinated by Rab35 such as cytokinesis and primary cilia formation are affected resulting in aberrant cell phenotypes.

The Rab GTPase superfamily shares a conserved G-domain specialized in GDP/GTP binding that allows the protein to switch between a GTP-bound active state and a GDP-bound inactive state (51). Structural analysis revealed that the R27H mutation is located near the G-domain; however, this residue does not interact directly with the nucleotide. Thus, the ability to bind GTP in this Rab35 variant is conserved. Conversely, its affinity toward the nucleotide becomes lower likely because of the structural destabilization of A151, a conserved residue from the G-domain, *via* the loss of a hydrogen bond between A151 and R27H. This prevents the protein from switching conformationally to its active form. It was shown that the interaction between Rab35 R27H and its GEFs, rather than transient becomes sustained, which can also affect the functioning of other Rabs that share these GEFs (9). In fact, the R27H mutation destabilizes the nucleotide binding by enhancing a spontaneous nucleotide dissociation, which confirms that Rab35 R27H is in an inactive conformation. More importantly, this Rab35 R27H variant exhibits low interaction with its effector, thus altering its biological function.

The role of Rab35 as a regulator of successful cytokinesis has been widely investigated (4, 17, 29). Subtle changes in the kinetics of cytokinesis or the clearance of midbody remnants could influence the fate of neural stem cell division during prenatal brain development (52). For instance, patients with a pathogenic missense variant in a gene involved in regulating cytokinesis exhibited intellectual disability and microcephaly (53). Recently, we identified that Rab35 is recruited to the cytokinetic bridge *via* its interaction with DENND2B, which functions as a GEF and effector for the GTPase (54), and promotes F-actin oxidation by Rab35 effector MICAL1 (30). It is likely that Rab35 R27H is not recruited to the cytokinetic bridge or is unable to promote successful cytokinetic abscission because of lack of interaction with effectors.

Several Rab GTPases have been associated with primary cilia formation and function, in either stimulatory roles or inhibitory roles (55). Primary cilia play critical roles in coordinating developmental and homeostatic signaling pathways. More importantly during neurodevelopment, primary cilia regulate the morphogenesis of embryonic cerebral cortex and cerebellum *via* the regulation of Shh and Wnt signaling pathways (56). Disruption of these ciliary functions seem to underlie a diverse spectrum of disorders. Rab35 regulates ciliary length and function and controls the ciliary membrane levels of Shh signaling regulators SMO, Arl13b, and INPP5E (18). In this study, we demonstrated that Rab35 R27H fails to localize to the ciliary membrane and that overexpressing this variant affects ciliary length, number, and Arl13b composition, in RPE-1 cells, suggesting that this Rab35 variant most likely disrupts Shh signaling, which is critical during spatial patterning of the neuroepithelium, axonal guidance, and neuronal activity (57). Among the major phenotypes exhibited by patients with ciliopathies, structural brain abnormalities, such as hydrocephalus, hypoplasia, cerebellar vermis hypoplasia, and Dandy–Walker malformation, stand as the more frequent ones (34). Consequently, ciliary defects caused by the Rab35 R27H variant could function as a major cause of the developmental disorder in the patient.

## Experimental procedures

The studies in this work abide by the Declaration of Helsinki principles. The human samples were obtained and analyzed under the approval of the Hadassah Medical Organization Institutional Review Board. Informed consent was obtained in accordance with Institutional Review Board–approved protocol 0306-10-HMO.

### Exome sequencing

Genomic DNA was extracted from peripheral blood samples of the proband and both parents. Exonic sequences were enriched in the DNA sample using the IDT xGen Exome Research Panel V2.0 capture combined with xGen Human mtDNA Research Panel v1.0 (Integrated DNA Technologies) and sequenced on a NovaSeq 6000 sequencing system (Illumina) as 100 bp paired-end runs. Data analysis including read alignment and variant calling was performed with DNAnexus software using default parameters, with the human genome assembly hg19/GRCh37 as reference. Variants were filtered out if they were off-target (intronic variants >8 bp from splice junction), synonymous (unless <4 bp from the splice site), or had minor allele frequency >0.01 in gnomAD or in our in-house exome database.

### Cell culture

Human embryonic kidney (HEK) 293T, HeLa, and RPE-1 cells were cultured using standard protocols. Cells were grown in Dulbecco's modified Eagle's medium (DMEM) with high glucose (GE Healthcare Life Sciences) supplemented with l-glutamine (Wisent Bioproducts), penicillin–streptomycin (Wisent Bioproducts), and 10% bovine calf serum (GE Healthcare Life Sciences) (regular media) at 37 °C in 5% CO_2_. Collection media for lentivirus production were prepared with regular medium supplemented with 1× nonessential amino acids and 1 mM sodium pyruvate. Starvation media for the cilia formation experiments contain DMEM with high glucose supplemented with l-glutamine and penicillin–streptomycin.

### Expression constructs

Complementary DNAs encoding full-length human Rab35 wt, Q67L, and S22N were previously described (13). For Rab35 overexpression lentivirus, mouse Rab35 was cloned in the pRRLsinPPT viral expression vector as previously described (22). Rab35 R27H was generated by site-directed mutagenesis (QuickChange Lightning kit; Agilent) using Rab35 wt constructs as template in the different backbone vectors and the following primers: 5′-CAAGAGCAGTTTACTGTTGCATTTTGCAGACAACACTTTC-3′; 5′-GAAAGTGTTGTCTGCAAAATGCAACAGTAAACTGCTCTTG-3′ for human Rab35; and 5′-CAAGAGCAGCTTGCTGTTGCATTTCGCAGACAACACCTTCTC-3′; 5′-GAGAAGGTGTTGTCTGCGAAATGCAACAGCAAGCTGCTCTTG-3′ for mouse Rab35. GST-GGA3 (amino acids 1–316) was generously provided by J. Bonifacino (National Institutes of Health). GST-MICAL1 (CC domain) was purchased from Synbio Technologies. All constructs were verified by sequence analysis.

### Lentivirus production

HEK 293T cells were seeded in 15 cm plates at the density of 1 × 10^7^. The cells were transfected with 12 μg of expression plasmid encoding the protein of interest, 6 μg of pMDLg/pRRE, 6 μg of pMD2.g, and 6 μg of pRSV-Rev using 1 mg/ml polyethylenimine (25 kDa). At 8 h post-transfection, the culture medium was replaced with collection media. The medium was collected at 24 and 36 h and stored at 4 °C until the last collection. Then it was filtered through a 0.45 μm membrane and concentrated by centrifugation (16 h at 6800 rpm). The resulting pellets were resuspended in DMEM in 1/5000 of the original volume, aliquoted, and stored at −80 °C until use.

The cells were transduced with multiplicity of infections of 5 on the day they were plated. The medium was replaced with fresh culture medium after 16 h. All experiments were performed between 3 and 15 days after transduction.

### Pull-down experiments

GST fusion proteins Rab35 wt/R27H/Q67L/S22N, MICAL1 CC domain, and GGA3 1 to 316 amino acids were expressed in *Escherichia coli* BL21. To purify Rab35 fusion protein and its variants, bacterial pellets were resuspended in 1× PBS supplemented with 5 mM MgCl_2_ and protease inhibitor cocktail. To purify MICAL1 CC domain and GGA3 1 to 316 amino acid fusion proteins, bacterial pellets were resuspended in 20 mM Tris–HCl buffer (pH 7.4), 150 mM NaCl, and 1 mM EDTA supplemented with protease inhibitor cocktail. Following sonication, Triton X-100 was added to the samples to a final concentration of 1% and then incubated for 30 min at 4 °C. Lysates were centrifuged for 15 min at 30,700*g*, and the supernatant was collected. The samples were then incubated with Glutathione Sepharose 4B (GE Healthcare Life Sciences) for 1 h at 4 °C, and the beads were washed with lysis buffer. Protein estimation was carried out by SDS-PAGE using a bovine serum albumin concentration curve.

Parallelly, FLAG-tagged CD1/CD2/Rab35 wt/Rab35 R27H/Rab35 Q67L/Rab35 S22N were expressed in HEK 293T cells. At 20 h post-transfection, cell lysates from FLAG-CD1/CD2-expressing cells were prepared in 20 mM Hepes, 100 mM NaCl_2_, 5 mM MgCl_2,_ and 1% Triton X-100. As for cells transfected with FLAG-Rab35/variants, lysates were prepared in 50 mM Tris–HCl buffer (pH 7.4), 100 mM NaCl_2_, 5 mM MgCl_2_, 0.5% Triton X-100, 0.25% deoxycholate, 0.05% SDS, and 5% glycerol. For Arf6 pull-down assay, HeLa cells transduced with GFP-Rab35 wt/GFP-Rab35 R27H were used, and lysates were prepared in 50 mM Tris–HCl buffer (pH 7.4), 150 mM NaCl, 10 mM MgCl_2_, 1% Triton, 0.5% deoxycholate, 0.1% SDS, and 5% glycerol. All lysis buffers were supplemented with protease inhibitors. The cell lysates were incubated for 30 min at 4 °C, centrifuged 232,000*g* for 15 min, and the supernatants were collected.

Pull-down experiments were performed using 50 μg of purified GST-fusion protein precoupled to Glutathione-Sepharose beads, and 1 mg of cell lysate except for Arf6 pull down for which we used 2 mg of lysates. The samples were incubated for 1 h at 4 °C and then washed with lysis buffer. Bound proteins were eluted in Laemmli buffer, resolved by SDS-PAGE, and processed for immunoblotting with the antibodies indicated; anti-FLAG (Sigma–Aldrich, F3165, 1:40,000 dilution), anti-Arf6 (Abcam, ab13126, 1:500 dilution), and anti-Actin (Abcam, ab6276, 1:5000 dilution).

### Nucleotide loading assay

GST-tagged Rab35 wt, S22N, and R27H were expressed in *E. coli* BL21. The fusion proteins were purified as described earlier and cleaved from GST tags with PreScission protease at 4 °C by overnight incubation. Cleaved GTPases were then exchanged into GEF loading buffer (20 mM Tris, pH 7.5, and 100 mM NaCl). Purified GTPases (0.4 μM) were incubated with 2.5 mM EDTA, 1.67 μCi [^35^S]GTPγS (PerkinElmer), and 2.5 μM GTPγS for 10 min at 30 °C in GEF loading buffer. Loaded nucleotides were then stabilized by the addition of 5 mM MgCl_2_. The reaction was then stopped by the addition of 1 ml cold wash buffer (20 mM Tris, pH 7.5, 100 mM NaCl, and 20 mM MgCl_2_) and passed through nitrocellulose filters to separate the nucleotide-bound proteins. The filters were washed with 5 ml wash buffer and counted using a liquid scintillation counter (Beckman Coulter LS6500 Liquid Scintillation Counter).

For the nucleotide dissociation assay, 0.4 μM of purified GTPases were loaded with nucleotide as described earlier. This reaction was performed at room temperature in 400 μl total volume. Loaded nucleotides were then stabilized by the addition of 5 mM MgCl_2_. Aliquots of 100 μl reaction were removed and added to 1 ml cold wash buffer containing 20 mM MgCl_2_ and incubated for indicated time points. Nucleotide-bound GTPases were passed through nitrocellulose filters. The filters were washed with 5 ml wash buffer and counted using a liquid scintillation counter (Beckman Coulter LS6500 Liquid Scintillation Counter).

### Cytokinesis assay

HeLa cells stably expressing GFP-Rab35 wt or R27H were seeded in glass bottom dishes (MatTek) with 60% confluency a day before the experiment. The cells were maintained at 37 °C and 5% CO_2_ and imaged every 10 min during 20 h, using Zeiss Observer.Z1 equipped with a Plan-Apochromat 10× objective (numerical aperture = 0.45) and a CCD camera (Axiocam 506 mono). Images were analyzed using ZEN 3.6 (blue edition) software.

### Cilia formation assay

RPE-1 cells were transduced with GFP-Rab35 wt or R27H and seeded in poly-l-lysine-coated coverslips at 70% confluency. After 16 h, the cells were incubated with starvation media for 24 h to induce cilia formation.

### Immunofluorescence staining

The cells were fixed in ice-cold methanol or 4% paraformaldehyde and processed for immunofluorescence using anti-CEP164 (Santa Cruz Biotechnology, sc-515403, 1:100 dilution) or anti-Arl13b (Proteintech, 66739-1-lg, 1:1000 dilution), respectively, and anti–ac-tubulin antibody (Cell Signaling Technology, D20G3, 1:1000 dilution), incubated 2 h at room temperature. Cells were washed with 1× PBS and incubated with corresponding Alexa Fluorophore–conjugated secondary antibody (Invitrogen, 1:250 dilution) for 1 h at room temperature. After washing, coverslips were mounted on a microscopic slide using fluorescence mounting medium (Dako). Imaging was performed using a 63× objective in a Leica SP8 laser scanning confocal microscope.

### Image processing

All images were analyzed using Fiji (ImageJ) (58). To determine cilia length, each cilium was manually traced using the segmented arrow tool. The degree of Rab35 localization to the ciliary membrane was determined *via* Pearson's correlation coefficient as a measure of colocalization between the fluorescent signals corresponding to Rab35 and ac-tubulin. This coefficient was obtained using JACoP plugin (59). The levels of Arl13b in the cilia were obtained by manually tracing each cilium as a region of interest, using the polygon tool, in the ac-tubulin channel, and overlying those regions of interest in the Arl13b channel to measure fluorescence. The images were prepared for publication using ImageJ. For better visualization, they were contrast-adjusted, and one pixel of Gaussian blur was applied. Final figures were assembled with Adobe Illustrator.

### Statistical analysis

Statistical analysis and graphs were made using GraphPad Prism software (GraphPad Software, Inc). Normality and lognormality tests were performed for all data. Normally distributed data were compared by using unpaired *t* test, as for nonnormally distributed data, comparisons were made using Mann–Whitney *U* test. All data are shown as means ± SEM, and the statistical significance represented as ∗*p* < 0.05; ∗∗*p* < 0.01; ∗∗∗*p* < 0.001; and ∗∗∗∗*p* < 0.0001.

## Data availability

All data are found in the article.

## Conflict of interest

The authors declare that they have no conflicts of interest with the contents of this article.
